# Endoscopic diagnosis of appendiceal hemorrhage with a disposable imaging catheter (EyeMax): a case report

**DOI:** 10.3389/fmed.2026.1734727

**Published:** 2026-02-18

**Authors:** Jiamu Zheng, Yuesheng Sun, Beibei Xu

**Affiliations:** 1 The Second Clinical Medical College of Zhejiang Chinese Medical University, Hangzhou, Zhejiang, China; 2 Department of General Surgery, Wenzhou Third Clinical Institute Affiliated to Wenzhou Medical University, Wenzhou People's Hospital, Wenzhou, Zhejiang, China; 3 Department of Gastroenterology, Wenzhou Third Clinical Institute Affiliated to Wenzhou Medical University, Wenzhou People's Hospital, Wenzhou, Zhejiang, China

**Keywords:** appendiceal hemorrhage, appendiceal ulceration, chronic appendicitis, EyeMax, visualization

## Abstract

**Background:**

Appendiceal hemorrhage is an extremely rare cause of lower gastrointestinal bleeding, often lacking specific clinical manifestations and therefore easily missed. Due to the anatomical location of the appendix, conventional colonoscopy often fails to visualize the appendiceal lumen directly, making diagnosis challenging.

**Case summary:**

A 53-year-old male was admitted with sudden onset of hematochezia. Emergency colonoscopy revealed blood throughout the colon without an identifiable bleeding source. Contrast-enhanced abdominal CT scan showed an enlarged appendix measuring approximately 10 mm in diameter, without signs of acute inflammation. As bleeding recurred the next day, a second colonoscopy was performed. A diverticulum in the cecum was suspected as the source and was clipped. However, while adjusting the scope, active bleeding was observed from the appendiceal orifice. In response, a disposable imaging catheter (EyeMax) was immediately advanced into the appendiceal lumen. The proximal lumen appeared clean, but deeper segments showed blood clots and ongoing bleeding, confirming appendiceal hemorrhage. The patient subsequently underwent laparoscopic appendectomy. Intraoperatively, the appendiceal lumen was filled with clots and active bleeding was observed. Gross examination of the specimen revealed a mucosal ulcer. Histopathology showed chronic appendicitis. The patient recovered well postoperatively, no further bleeding occurred during 6-month follow-up.

**Conclusion:**

The use of disposable imaging catheters enables direct endoscopic visualization of appendiceal intraluminal pathology, thereby improving diagnostic precision in challenging appendiceal cases. This minimally invasive technique shows promising clinical utility and may serve as a valuable adjunct in the diagnostic algorithm of rare appendiceal bleeding disorders.

**Core Tip:**

This case demonstrates the utility of a disposable imaging catheter in identifying active appendiceal bleeding, which is often missed by conventional endoscopy. The technique offers a novel diagnostic option for this rare but important condition.

## Introduction

Appendiceal hemorrhage is an exceptionally rare clinical entity that presents significant diagnostic challenges due to its concealed anatomical location and non-specific clinical manifestations (1, 2). The source of bleeding is often misattributed to the terminal ileum or proximal colon, given the proximity of the appendix to the ileocecal valve and the limitations of conventional colonoscopy in directly visualizing the appendiceal lumen (1). In recent years, advances in endoscopic technology have enabled more precise evaluation of obscure lower gastrointestinal bleeding sources. Among these, catheter-based imaging techniques offer a promising opportunity to enhance diagnostic accuracy in selected patients (3, 4). Herein, we present a case of appendiceal hemorrhage caused by mucosal ulceration in the context of chronic appendicitis, successfully diagnosed using a disposable imaging catheter inserted under colonoscopic guidance. This report highlights the evolving role of novel endoscopic tools in the diagnosis of rare but clinically significant appendiceal disorders.

## Case presentation

A 53-year-old male presented with hematochezia for 1 day. He reported the onset of hematochezia at home, without any apparent precipitating factors or associated abdominal pain. His medical history was notable only for hypertension for 5 years, but he was taking no medications. He denied a history of diabetes, cardiovascular or cerebrovascular diseases, gastrointestinal disorders, non-steroidal anti-inflammatory drug (NSAID) use, infectious diseases or previous surgeries. There was no relevant personal or family history.

On physical examination, the patient appeared mildly anemic, the abdomen soft and non-tender, without tenderness or signs of peritoneal irritation. The perianal area was unremarkable. Digital rectal examination revealed no palpable mass within reach, but the glove was stained with dark red blood. Laboratory evaluation revealed anemia without evidence of coagulation abnormalities.

**Figure 1:**
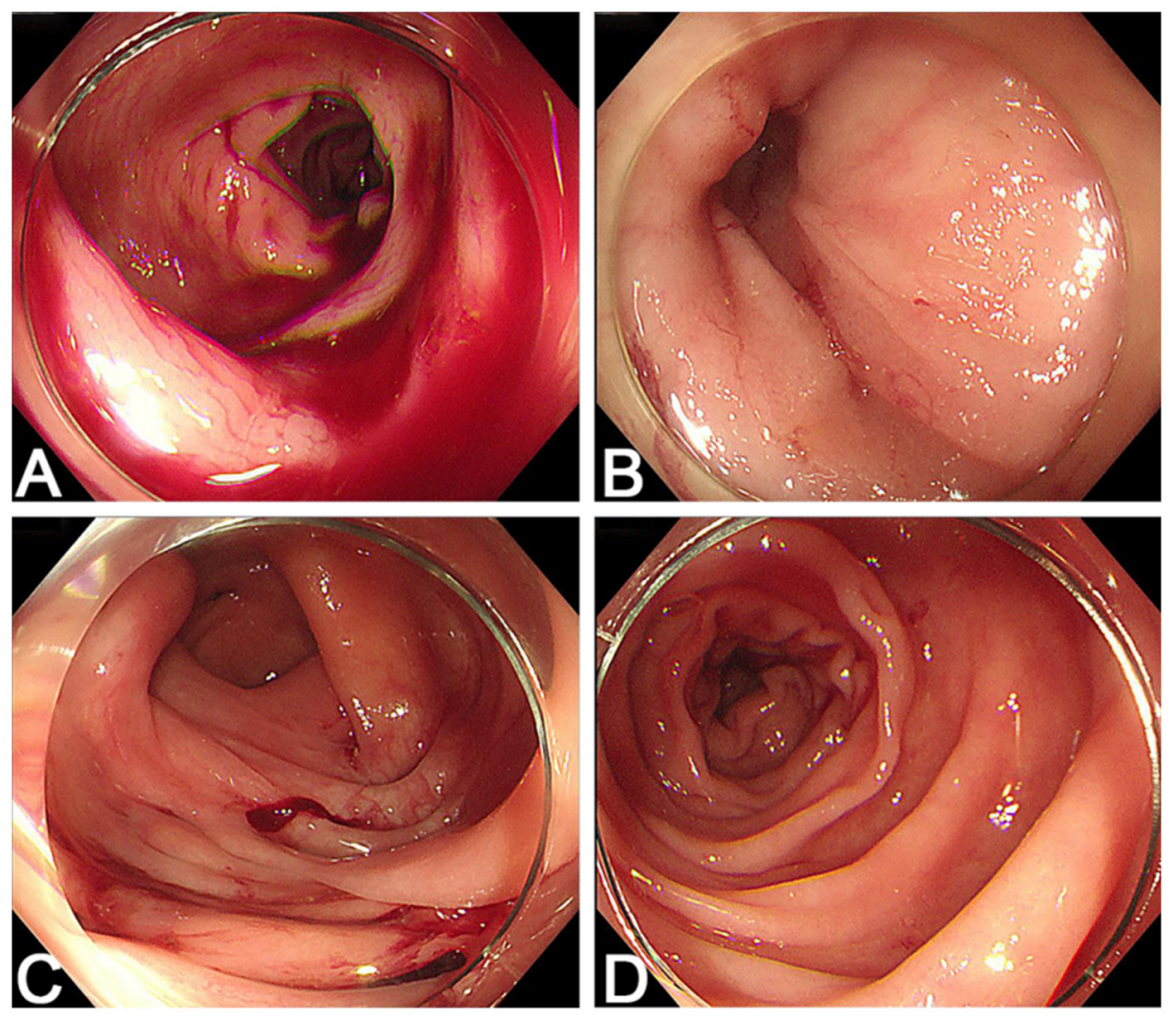
Initial colonoscopy performed on admission. **(A)** A large amount of fresh blood is seen in the colonic lumen. **(B)** The terminal ileum appears normal, with no signs of bleeding. **(C)** Multiple diverticula are observed in the cecum and ascending colon. **(D)** After irrigation, no active bleeding source is identified.

**Figure 2:**
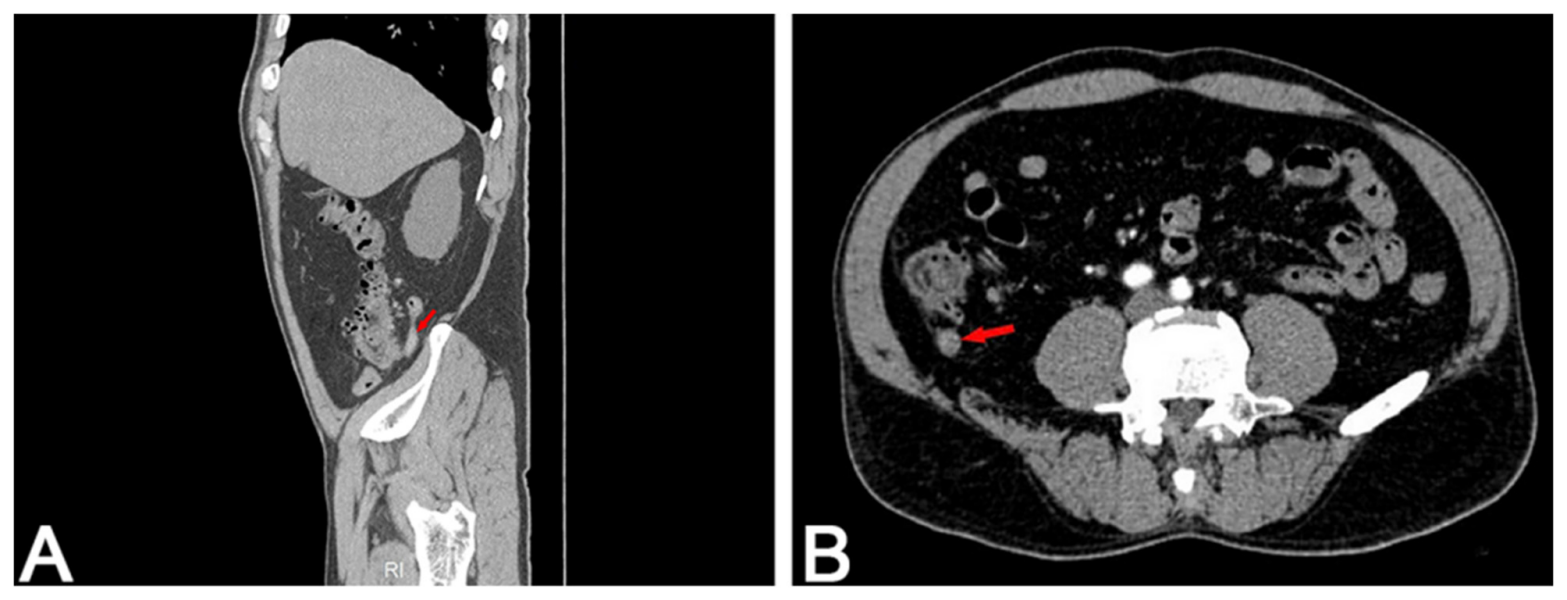
Contrast-enhanced abdominal CT scan. **(A)** Sagittal reconstruction shows an enlarged appendix with a maximal diameter of approximately 10 mm. **(B)** Axial view reveals a thickened appendix without evidence of acute appendicitis or surrounding fat stranding.

An emergency colonoscopy was performed after admission, which revealed blood in the colonic lumen but no definitive bleeding source (Figures 1A–D). Contrast-enhanced abdominal CT scan showed an enlarged appendix measuring approximately 10 mm in diameter (Figure 2).

**Figure 3:**
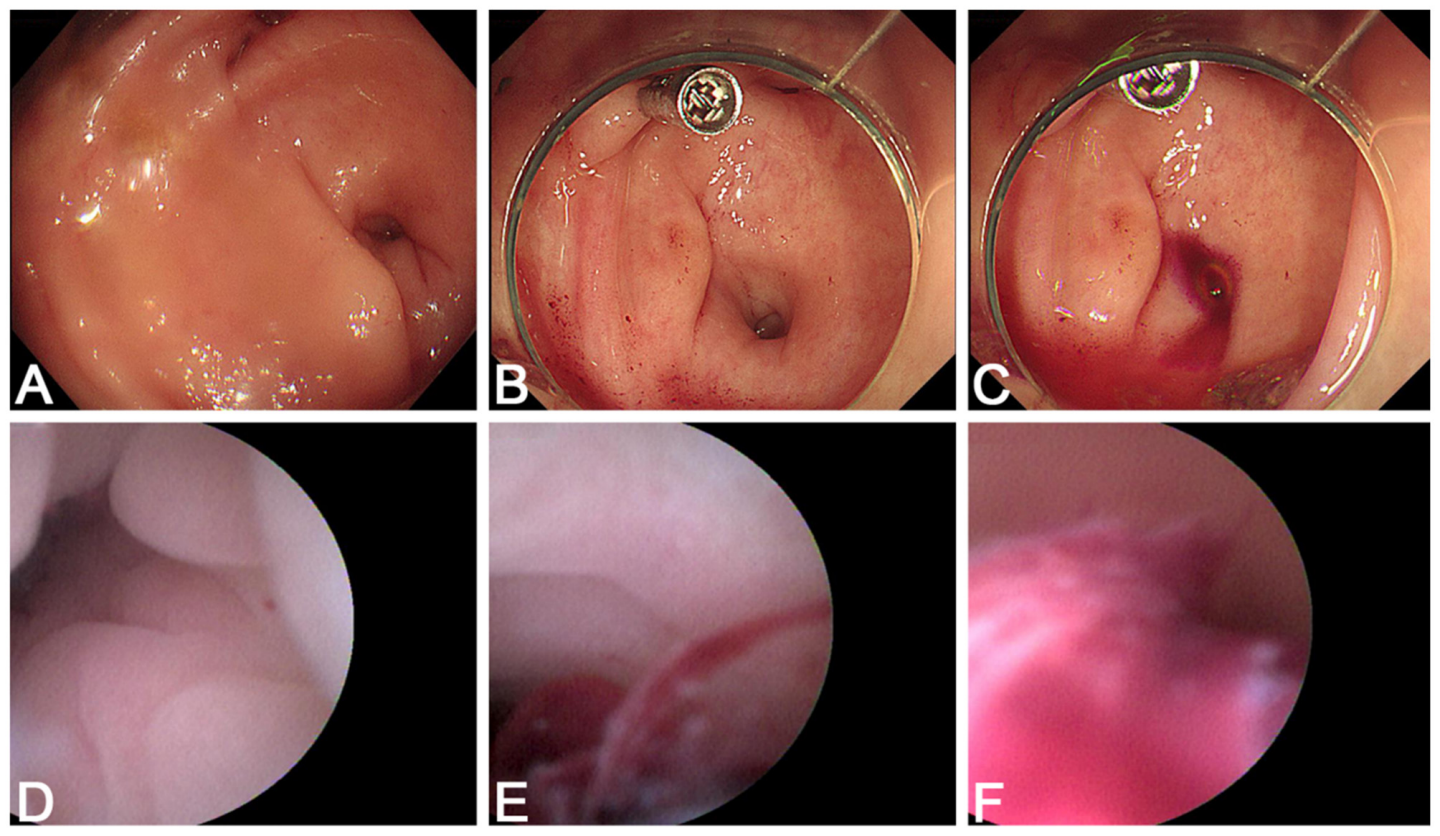
Second emergency colonoscopy with appendiceal catheterization. **(A)** After irrigation, a few traces of blood are seen near the cecal diverticula, raising suspicion of diverticular bleeding. **(B)** A hemostatic clip is placed on the suspected bleeding diverticulum. **(C)** Shortly after clip placement, fresh blood is seen actively oozing from the appendiceal orifice. **(D)** A disposable imaging catheter (EyeMax) is inserted into the appendix; no obvious bleeding is seen at the entry. **(E)** As the catheter advances, blood becomes visible within the appendiceal lumen. **(F)** A large amount of blood and clots fill the distal lumen, confirming active appendiceal bleeding.

**Figure 4:**
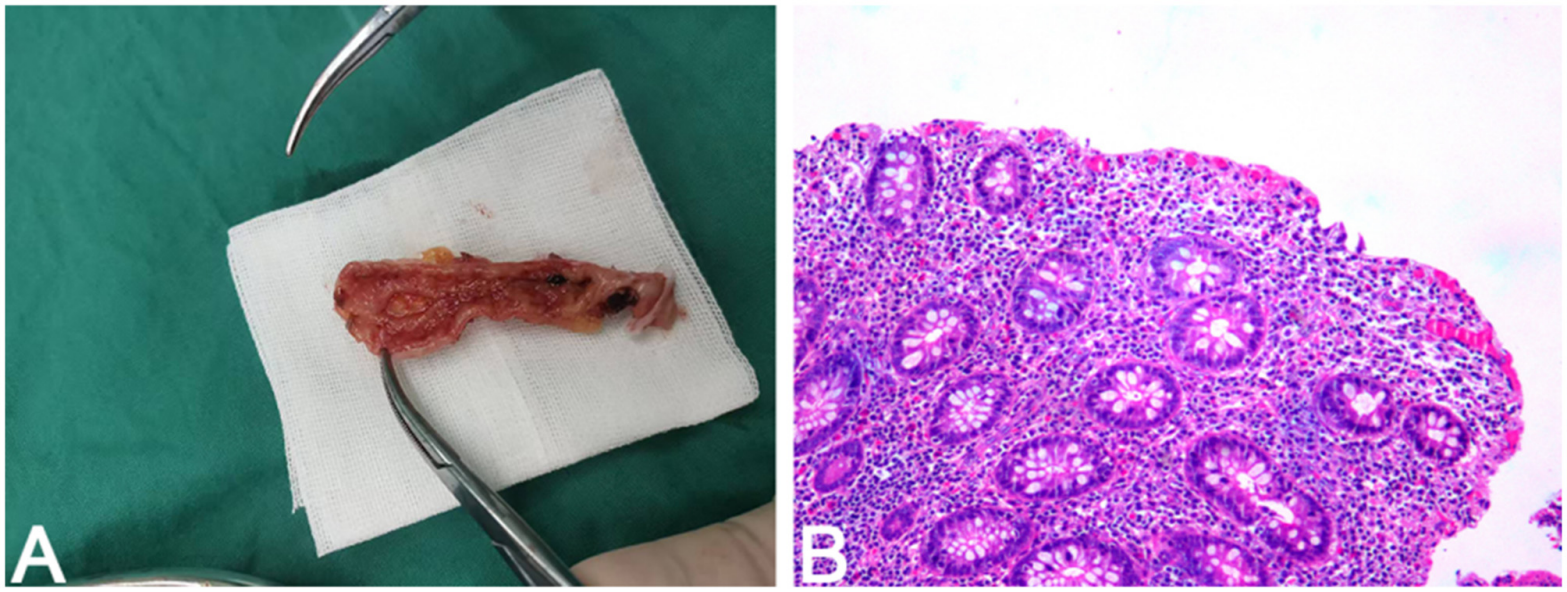
Gross and histopathological findings of the resected appendix. **(A)** Gross specimen of the resected appendix opened longitudinally, showing a focal mucosal defect suggestive of superficial ulceration. **(B)** Histological section of the appendix (H&E, 100 × ) reveals lymphoplasmacytic infiltration and architectural distortion of the mucosal glands, consistent with chronic appendicitis. The ulcer site was not captured in the sampled section.

The following day, the patient experienced another episode of hematochezia, with hemoglobin dropping from 94 g/L to 79 g/L(reference range: 130–175 g/L), considering the existence of active bleeding. A repeat colonoscopy was performed. During the second colonoscopy, numerous diverticula were noted in the cecum and ascending colon. After thorough irrigation, blood was seen in the cecal lumen, and one diverticulum was initially suspected and clipped. However, while adjusting the scope, a sudden gush of fresh blood was observed from the appendiceal orifice (Figures 3A–C). In response, a disposable imaging catheter (EyeMax; Micro-Tech, Nanjing, China; 9F) was immediately advanced into the appendiceal lumen. Intraluminal inspection demonstrated a large number of blood clots and active hemorrhage, thereby confirming the true source of hemorrhage (Figures 3D–F). The patient was subsequently referred to the surgical department and underwent laparoscopic appendectomy. Intraoperatively, the appendiceal lumen was filled with blood clots and active bleeding was observed. Gross examination of the resected specimen revealed superficial ulceration of the appendiceal mucosa (Figure 4A). Histopathological analysis confirmed chronic appendicitis (Figure 4B). Based on the clinical, endoscopic, and histopathological findings, the final diagnosis was chronic appendicitis with appendiceal hemorrhage.

The postoperative course was uneventful. No further episodes of hematochezia or melena occurred, and no recurrent gastrointestinal bleeding was observed during follow-up. At 6 months after surgery, the patient remained asymptomatic, with no evidence of rebleeding.

## Discussion

Appendiceal hemorrhage is a rare and frequently overlooked cause of lower gastrointestinal bleeding, with an estimated incidence of less than 0.02% (2). The underlying pathogenesis remains incompletely understood. Reported etiologies include appendiceal ulceration, vascular malformations, acute or chronic appendicitis, neoplasms, fecaliths, and diverticula (5–11).

Due to the concealed anatomical location of the appendix adjacent to the ileocecal valve, appendiceal bleeding is often misattributed to the small intestine or proximal colon (1). Although CT and conventional colonoscopy may suggest bleeding in the appendiceal region, they are unable to provide intraluminal confirmation. Accurate diagnosis is further complicated by the limited ability of conventional colonoscopy to assess the appendiceal lumen, particularly in patients without typical appendicitis symptoms (3, 7). Several endoscopic technologies, including the SpyGlass system and ultrathin endoscopes, have been reported for appendiceal visualization, however, their clinical application remains limited by technical complexity, high cost, and limited field of view (3, 4). Endoscopic hemostasis using colonoscopic clipping has also been described in selected cases of appendiceal hemorrhage (12–14). Previous case reports and literature reviews have summarized the clinical experience in managing this rare condition (15–17). Despite these reports, direct intraluminal visualization of the appendix remains challenging in routine practice, and clinical experience with simpler, catheter-based imaging tools is still limited.

In the present case, a disposable imaging catheter (EyeMax) was successfully inserted into the appendiceal lumen under colonoscopic guidance. A large volume of blood clots and active bleeding was directly visualized, allowing for precise localization and definitive diagnosis. The clinical utility of this approach was thereby demonstrated in resolving obscure cases of gastrointestinal bleeding. Chronic appendicitis with mucosal ulceration was confirmed by postoperative histopathological examination. No signs of acute inflammation were observed during the clinical course, suggesting that ulcer formation and subsequent hemorrhage may have resulted from long-standing mucosal injury and localized ischemia. This mechanism has been infrequently reported in the literature and remains a diagnostic challenge in clinical practice.

With the growing availability of catheter-assisted imaging tools, the direct assessment of the appendiceal lumen is expected to serve as a valuable adjunct to conventional endoscopy (4). This strategy may enhance diagnostic precision in patients with suspected appendiceal pathology, particularly when classical symptoms are absent.

Furthermore, the broader implementation of minimally invasive visualization techniques may facilitate earlier detection of rare appendiceal pathologies and improve clinical decision-making. Although the disposable imaging catheter adds procedural cost, its targeted use in selected patients may be cost-effective by enabling precise intraluminal localization and reducing unnecessary diagnostic procedures or delays in definitive management. Future prospective studies and larger case series are warranted to further validate the diagnostic accuracy, safety profile, and cost-effectiveness of this approach in routine endoscopic practice.

Currently, EyeMax functions exclusively as a diagnostic imaging catheter, as no compatible endoscopic hemostatic accessories are available. In the present case, its role was limited to diagnostic clarification rather than endoscopic treatment. Nevertheless, direct intraluminal visualization of the appendiceal lumen enabled accurate localization of the bleeding source and supported subsequent management decisions.

## Conclusion

This case demonstrates that disposable imaging catheters can facilitate direct intraluminal visualization of the appendix and improve diagnostic accuracy in selected cases of obscure lower gastrointestinal bleeding. When applied selectively, this technique may help avoid repeated or inconclusive diagnostic procedures and support timely clinical decision-making, thereby offering potential cost-effectiveness in clinical practice.

## Data Availability

The original contributions presented in the study are included in the article/supplementary material, further inquiries can be directed to the corresponding author.
